# Efficacy and safety of zuranolone in Japanese adults with major depressive disorder: A double‐blind, randomized, placebo‐controlled, phase 2 clinical trial

**DOI:** 10.1111/pcn.13569

**Published:** 2023-06-21

**Authors:** Masaki Kato, Kazuyuki Nakagome, Takamichi Baba, Takuhiro Sonoyama, Daiki Okutsu, Hideki Yamanaka, Ryosuke Shimizu, Tomoko Motomiya, Takeshi Inoue

**Affiliations:** ^1^ Department of Neuropsychiatry Kansai Medical University Osaka Japan; ^2^ Department of Psychiatry National Center of Neurology and Psychiatry Tokyo Japan; ^3^ Biostatistics Center, Drug Development and Regulatory Science Division Shionogi & Co., Ltd. Osaka Japan; ^4^ Medical Science Department, Drug Development and Regulatory Science Division Shionogi & Co., Ltd. Osaka Japan; ^5^ Clinical Research Department, Drug Development and Regulatory Science Division Shionogi & Co., Ltd. Osaka Japan; ^6^ Clinical Pharmacology & Pharmacokinetics, Drug Development and Regulatory Science Division Shionogi & Co., Ltd. Osaka Japan; ^7^ Project Management Department, Drug Development and Regulatory Science Division Shionogi & Co., Ltd. Osaka Japan; ^8^ Department of Psychiatry Tokyo Medical University Tokyo Japan

**Keywords:** depressive disorder, Japan, phase 2, safety, zuranolone

## Abstract

**Aim:**

To evaluate the efficacy and safety of an oral, once‐daily, 14‐day treatment course of zuranolone in Japanese patients with major depressive disorder (MDD).

**Methods:**

This multicenter, randomized, double‐blind, placebo‐controlled study randomized eligible patients (1:1:1) to receive oral zuranolone 20 mg, zuranolone 30 mg, or placebo once daily for 14 days (treatment‐period), followed by two 6‐week follow‐up periods. The primary endpoint was change from baseline in the 17‐item Hamilton Depression Rating Scale (HAMD‐17) total score on Day 15.

**Results:**

Overall, 250 patients (enrolled: 07/07/2020–05/26/2021) were randomized to receive placebo (*n* = 83), zuranolone 20 mg (*n* = 85), or zuranolone 30 mg (*n* = 82). The demographic and baseline characteristics were balanced between groups. The adjusted mean (standard error) change from baseline in the HAMD‐17 total score on Day 15 was −6.22 (0.62), −8.14 (0.62), and − 8.31 (0.63) in the placebo, zuranolone 20‐mg, and zuranolone 30‐mg groups, respectively. Significant differences in the adjusted mean (95% confidence interval [CI]) for zuranolone 20 mg versus placebo (−1.92; [−3.65, −0.19]; *P* = 0.0296) and zuranolone 30 mg versus placebo (−2.09; [−3.83, −0.35]; *P* = 0.0190) groups were observed on Day 15, and also as early as Day 3. A nonsignificant yet distinct drug‐placebo separation was observed during follow‐up. Somnolence (placebo [3.7%], zuranolone 20 mg [10.6%], and zuranolone 30 mg [20.7%]) and dizziness (3.7%, 9.4%, and 9.8%, respectively) were more common with zuranolone.

**Conclusion:**

Oral zuranolone was safe and demonstrated significant improvements in depressive symptoms, as assessed by HAMD‐17 total score change from baseline over 14 days in Japanese patients with MDD.

According to the World Health Organization estimates, depression is a common illness worldwide, affecting approximately 3.8% of the global population, including 5.0% of adults.^1^ The Global Burden of Diseases, Injuries, and Risk Factors Study 2019 reported depressive disorders as the 19th leading cause of disability‐adjusted life‐years in all age groups and the eighth leading cause among younger generations aged between 10 and 49 years.^2^ Further, the global prevalence or burden of depressive disorders continues to increase despite available treatment.^3^

Globally, depression is among the largest contributors of work performance loss, absenteeism, and economic impact on the employer.^3^, ^4^ The odds of remission decrease with every subsequent line of treatment.^5^ Additionally, the time to response to existing antidepressants is usually long (weeks to months),^6^, ^7^, ^8^, ^9^, ^10^ thereby necessitating long‐term treatment to achieve remission.

When an antidepressant is ineffective for a long period of time, the risk of prolonged or aggravated symptoms of debilitation, including the risk of suicide, increases.^11^ Even when an antidepressant is effective, maintaining long‐term treatment adherence is challenging.^12^, ^13^, ^14^, ^15^

Depression is also prevalent in Japan, with approximately 6% of Japanese likely to experience major depressive disorder (MDD) at least once in their lifetime.^16^ Overall, the level of satisfaction with the current treatment for depression is still insufficient, and new therapeutic drugs are desired.^17^

To this end, an oral synthetic neuroactive steroid, zuranolone, was developed (SAGE‐217 in the United States [US] and S‐812217 in Japan) by modification of the endogenous neuroactive steroid allopregnanolone. Zuranolone, a positive allosteric modulator of synaptic and extrasynaptic γ‐aminobutyric acid type A (GABA_A_) receptors (similar to allopregnanolone), is in clinical development as an oral, once‐daily, 14‐day treatment course for adults with MDD and postpartum depression.^18^

Several preclinical studies have suggested that allopregnanolone may exert an antidepressant effect by reducing the physiological effects of stress, promoting neuroprotection, suppressing proinflammatory immune activation associated with depression, and protecting against cytokine hypersecretion.^19^, ^20^, ^21^ Moreover, patients with depression have a reduced level of allopregnanolone in the cerebrospinal fluid, which returns to normal levels once the antidepressant is administered.^22^, ^23^ Consequently, based on the favorable outcome of a clinical study conducted in patients with postpartum depression, injectable allopregnanolone (brexanolone) was approved in the US for the treatment of postpartum depression in adults.^24^

Zuranolone, which has a physiological activity similar to that of allopregnanolone, may exert an antidepressant effect by enhancing the GABA_A_ receptor function. Important evidence for the antidepressant mechanism hypothesis of zuranolone includes decreased allopregnanolone levels observed in major depression,^22^ abundant distribution of allopregnanolone in the amygdala,^25^ and an antidepressant effect mediated by enhancement of amygdala theta (θ) activity by enhancement of extrasynaptic GABA receptor function on inhibitory neurons in the amygdala.^26^ Several studies hypothesize that existing antidepressants such as selective serotonin reuptake inhibitors (SSRIs) and serotonin and norepinephrine inhibitors (SNRIs) take time to develop effects^27^; however, their mechanisms have not been fully elucidated. We speculate that zuranolone supplements the weakened GABA‐enhancing effect of allopregnanolone, and is thought to exert a rapid‐acting antidepressant effect through a mechanism of action different from that of existing antidepressants. The overall efficacy and safety of zuranolone (up to 50‐mg once‐daily dose) have been demonstrated in phase 2 and 3 clinical trials conducted in patients with MDD.^28^, ^29^, ^30^, ^31^, ^32^, ^33^ The 14‐day treatment regimen of zuranolone demonstrated a rapid onset of activity with clinically meaningful improvements in depressive symptoms, which may offer the potential to treat MDD episodically as needed.^29^, ^30^, ^31^, ^32^, ^33^

In Japan, a phase 1 study showed no major safety and tolerability issues (Sonoyama *et al*., article under submission).

This study aims to evaluate the efficacy, safety, and pharmacokinetics (PKs) of zuranolone 20 mg and 30 mg once daily for 14 days in Japanese patients with MDD. This study is expected to provide information for the development of the study design for phase three and subsequent studies.

## Methods

### Study design

**Fig. 1:**
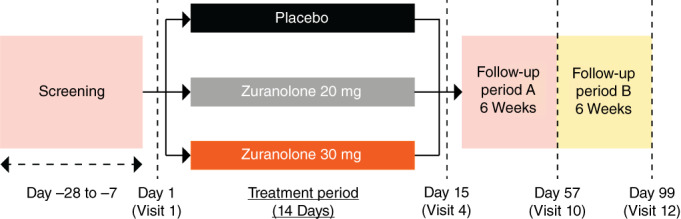
Study design.

This multicenter, randomized, double‐blind, placebo‐controlled, parallel‐group study was conducted in patients with MDD at 72 sites in Japan (JapicCTI‐205276). After screening for eligibility and obtaining informed consent (Days −28 to −7), patients were randomized (1:1:1) at baseline (visit 1), with stratification based on the 17‐item Hamilton Depression Rating Scale (HAMD‐17) total score at baseline (<25 vs ≥25) and sex, to receive zuranolone 20 mg, zuranolone 30 mg, or matching placebo once daily for 14 days (double‐blind treatment period). The study period comprised a 14‐day double‐blind treatment period, followed by two 6‐week follow‐up periods, A and B. During the treatment period, patients were followed up on Days 3 (visit 2), 8 (visit 3), and 15 (visit 4). During period A, patients were followed up weekly for 6 weeks (visit 5 [Day 22] to visit 10 [Day 57]) without treatment with the study drug. During period B, patients were followed up on a voluntary basis on Days 71 (visit 11) and 99 (visit 12) without treatment with the study drug (Fig. 1).

This study was approved by the institutional review board and the ethics committee at every study site. This study was conducted in accordance with the Declaration of Helsinki and Council for International Organizations of Medical Sciences' International Ethical Guidelines, applicable International Council for Harmonization of Technical Requirements for Pharmaceuticals for Human Use, Good Clinical Practice Guidelines, and other applicable laws and regulations. Written informed consent was obtained from all patients or legally acceptable representatives.

### Patients

Japanese patients aged between ≥18 years and ≤ 75 years at the time of signing the informed consent form (ICF), with MDD diagnosis evaluated using Mini‐International Neuropsychiatric Interview (M.I.N.I.; according to the Diagnostic and Statistical Manual of Mental Disorders, fifth edition [DSM‐5]) were eligible to participate if their current MDD episode was ongoing for at least 8 weeks and ≤ 12 months prior to signing the ICF.

Key exclusion criteria included evidence of serious comorbid medical conditions, treatment‐resistant depression (TRD) (no improvement in depressive symptoms with at least two different antidepressants [excluding antipsychotics] for an existing depressive episode at adequate approved doses for 4 weeks and using the Massachusetts General Hospital Antidepressant Treatment Response Questionnaire), use of antidepressants within 14 days prior to Day 1 (baseline/visit 1), and risk of suicide. The full list of inclusion/exclusion criteria is provided in Table S1.

### Intervention

Patients in the zuranolone 30‐mg group received three 10‐mg capsules once daily for 14 days; those in the zuranolone 20‐mg group received two 10‐mg capsules daily and one matching placebo for 14 days; and those in the placebo group received three matching placebo capsules for 14 days. All capsules were to be taken within 1 h after an evening meal or with a light meal before bedtime if the former was not possible.

The study drug assignment table was maintained in the Interactive Web Response System throughout the double‐blind treatment period. The sponsor's personnel were unblinded after the data of all patients were locked by completion of follow‐up period A (up to visit 10). Patient discontinuation criteria such as liver chemistry stopping criteria, corrected QT interval (QTc) stopping criteria, discontinuation of the study drug due to pregnancy, discontinuation of the study drug for other reasons, patient discontinuation/withdrawal from the study, lost to follow‐up, and prior and concomitant therapy are presented in Table S2.

### Endpoints

The primary endpoint was change from baseline (visit 1) in the HAMD‐17 total score on Day 15 (visit 4). Secondary endpoints included treatment response (≥50% reduction from baseline in HAMD‐17 total score) and remission (HAMD‐17 score of ≤7); and change from baseline in HAMD‐17 subscale scores. The core symptoms (subscale) contain assessment of depressed mood, feeling of guilt, suicide, and retardation. The anxiety subscale contains assessment of psychiatric/psychic anxiety, somatic anxiety, gastrointestinal somatic symptoms, general somatic symptoms, hypochondriasis, and insight. Bech‐6 subscale contains assessment of depressed mood, feeling of guilt, work and activities, psychiatric/psychic anxiety, general somatic symptoms, and retardation. Maier subscale contains assessment of depressed mood, feeling of guilt, work and activities, agitation, psychiatric/psychic anxiety, and somatic anxiety. The insomnia symptoms subscale contains assessment of initial insomnia, insomnia during the night, and delayed insomnia. Other secondary endpoints include Patient Global Impression of Improvement (PGI‐I) scores; improvement in Clinical Global Impression–Severity of Illness (CGI‐S) score; change from baseline in the 9‐item Patient Health Questionnaire (PHQ‐9) score; change from baseline in 8‐domain scores and summary scores of the 36‐item Short Form Health Survey (SF‐36); change from baseline in insomnia severity index (ISI) total score; incidence of adverse events (AEs)/treatment‐related AEs evaluated throughout the study period; and plasma zuranolone concentration.

### Statistical analysis

Full analysis set (FAS) comprised all patients randomly assigned to the study drug and administered at least one dose of the study drug and had their HAMD‐17 scores measured at baseline and at least one time point after treatment initiation. FAS was used for the primary analysis of the primary and secondary efficacy endpoints. PK analysis set comprised all patients who received at least one dose of zuranolone with at least one evaluable concentration of zuranolone in plasma. Safety analysis set (SAS) comprised all patients randomly assigned to the study drug who took at least one dose of the study drug and underwent at least one safety assessment. All statistical tests were performed at a two‐sided significance level of 0.05 unless otherwise specified. Only in the primary analysis of the primary efficacy endpoint, a fixed sequence procedure was used to prevent inflation of type 1 error due to multiple testing. Particularly, the comparison of the primary endpoint between the zuranolone 30‐mg and placebo groups was performed at a two‐sided significance level of 0.05, and only when significance was achieved, the comparison of the primary endpoint between the zuranolone 20‐mg and placebo groups was performed at a two‐sided significance level of 0.05.

In the primary analysis, the change from baseline in the HAMD‐17 total score on Day 15 was compared between the zuranolone 20‐ or 30‐mg group and placebo group using a mixed‐effects model for repeated measures (MMRM), assuming that the missing data were ‘missing at random.’ Using all available data obtained on Days 3–57, MMRM was applied, with the change from baseline as the response variable; the intervention group, time point, and interaction between the intervention group and time point as fixed effects; and the HAMD‐17 total score at baseline and sex as covariates. Sample size estimation and details of MMRM are presented in Table S1.

Inverse probability‐weighted generalized estimating equation (IPW‐GEE)^34^ was used to compare HAMD‐17 response and remission and response of CGI‐S, and PGI‐I scores. Using all available data obtained on Days 3–57, IPW‐GEE was applied, with the intervention group, time point, and interaction between the intervention group and time point as fixed effects and the score at baseline and sex as covariates. IPW‐GEE assumed an independent structure for the working correlation matrix. Details of the weight of IPW‐GEE^34^ are presented in Table S1. The data at the earliest time point were excluded to avoid non‐convergence. For PGI‐I, because there are no baseline values, the baseline value of the CGI‐S was used as a covariate in IPW‐GEE.

The plasma zuranolone concentrations were presented as a scatter plot on Day 8 (visit 3) and Day 15 (visit 4). AEs were classified by system organ class and preferred term according to Medical Dictionary for Regulatory Activities (MedDRA) Version 23.0 update. Among AEs reported in electronic case report forms (eCRFs), treatment‐emergent AEs (TEAEs) were used for safety analyses. A TEAE was defined as any AE with an onset date on or after the first dose of the study drug. TEAEs leading to death, serious TEAEs other than death, TEAEs of special interest (all events related to sedation, somnolence, dizziness, respiratory failure, and suspected to be drug dependence [events classified as drug abuse and dependence in the International Council for Harmonization of Technical Requirements for Pharmaceuticals for Human Use MedDRA, by the Standardized MedDRA Queries]), and TEAEs leading to treatment discontinuation were summarized. TEAEs assessed as related to the study drug were regarded as treatment‐related AEs. All analyses were performed using SAS, version 9.4.

## Results

### Patient disposition

A total of 250 patients were enrolled between 7 July 2020, and 26 May 2021, and randomized (1:1:1) to receive placebo (*n* = 83), zuranolone 20 mg (*n* = 85), or zuranolone 30 mg (*n* = 82).

**Fig. 2:**
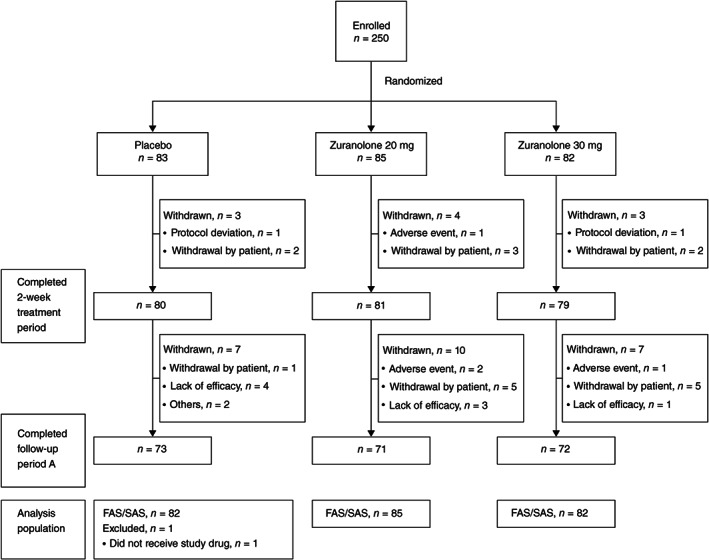
CONSORT flow chart. Abbreviations: FAS, full analysis set; SAS, safety analysis set.

Of the 250 patients, 240 completed the 14‐day treatment period (placebo [*n* = 80]; zuranolone 20 mg [*n* = 81]; zuranolone 30 mg [*n* = 79]). A similar proportion of patients withdrew during the treatment period across the groups: 3.6% of patients in the placebo group, 4.7% in the zuranolone 20‐mg group, and 3.7% in the zuranolone 30‐mg group; the most common reason for withdrawal was ‘withdrawal by patient’ during the treatment period (Fig. 2: CONSORT flow chart).

Two hundred sixteen patients (placebo [*n* = 73]; zuranolone 20 mg [*n* = 71]; zuranolone 30 mg [*n* = 72]) completed the follow‐up period A (Day 57). The most common reason for withdrawal during this follow‐up period A was ‘lack of efficacy’ in the placebo group and ‘withdrawal by patient’ in the zuranolone 20‐mg and 30‐mg groups (Fig. 2: CONSORT flow chart).

Over 80% of the randomized patients (*n* = 206: placebo [*n* = 69]; zuranolone 20 mg [*n* = 69]; zuranolone 30 mg [*n* = 68]) proceeded to the follow‐up period B, of which 200 patients (placebo [*n* = 67]; zuranolone 20 mg [*n* = 65]; zuranolone 30 mg [*n* = 68]) completed the follow‐up period B. Some patients did not proceed to (*n* = 4, *n* = 2, *n* = 4) or withdrew from (*n* = 2, *n* = 4, *n* = 0) follow‐up period B in the placebo, zuranolone 20‐mg, and zuranolone 30‐mg groups, respectively. The reasons for not having proceeded to or for having withdrawn from follow‐up period B were withdrawal by patients, lack of efficacy, or lost to follow‐up. The FAS/SAS comprised 82 patients in the placebo group, 85 in the zuranolone 20‐mg group, and 82 in the zuranolone 30‐mg group. One patient in the placebo group did not receive the study drug and was excluded from the SAS and FAS (Fig. 2: CONSORT flow chart).

### Demographics and baseline characteristics

Overall, the demographics and baseline characteristics (mean [standard deviation (SD)]), including age (39.6 [11.7] years) and baseline HAMD‐17 total score (24.6 [2.2]) were similar across the treatment groups (Table 1).

**Table 1:** Demographics and baseline characteristics (FAS)

	Placebo *N* = 82 *n* (%)	Zuranolone 20 mg *N* = 85 *n* (%)	Zuranolone 30 mg *N* = 82 *n* (%)	Total *N* = 249 *n* (%)
Sex				
Male	35 (42.7)	36 (42.4)	35 (42.7)	106 (42.6)
Female	47 (57.3)	49 (57.6)	47 (57.3)	143 (57.4)
Age (years)				
≥18 to <25	7 (8.5)	11 (12.9)	13 (15.9)	31 (12.4)
≥25 to <45	42 (51.2)	41 (48.2)	41 (50.0)	124 (49.8)
≥45 to <65	33 (40.2)	32 (37.6)	27 (32.9)	92 (36.9)
≥65	0	1 (1.2)	1 (1.2)	2 (0.8)
Mean (SD)	40.8 (10.6)	39.3 (12.6)	38.8 (12.0)	39.6 (11.7)
Height (cm)				
Mean (SD)	163.7 (7.5)	163.9 (7.6)	163.6 (8.9)	163.7 (8.0)
Weight (kg)				
Mean (SD)	63.4 (16.5)	64.6 (13.8)	61.0 (12.9)	63.0 (14.5)
BMI (kg/m^2^)				
Mean (SD)	23.6 (5.3)	23.9 (4.4)	22.7 (4.0)	23.4 (4.6)
Race (ethnicity)				
Asian	82 (100.0)	85 (100.0)	82 (100.0)	249 (100.0)
Not Hispanic or Latino	82 (100.0)	85 (100.0)	82 (100.0)	249 (100.0)
Employment status				
Full‐time (≥35 h per week)	30 (36.6)	28 (32.9)	22 (26.8)	80 (32.1)
Part‐time (<35 h per week)	13 (15.9)	11 (12.9)	14 (17.1)	38 (15.3)
Unemployed	10 (12.2)	19 (22.4)	21 (25.6)	50 (20.1)
Retired	9 (11.0)	4 (4.7)	5 (6.1)	18 (7.2)
Other	20 (24.4)	23 (27.1)	20 (24.4)	63 (25.3)
Concurrent disease				
Yes	65 (79.3)	65 (76.5)	50 (61.0)	180 (72.3)
No	17 (20.7)	20 (23.5)	32 (39.0)	69 (27.7)
Baseline value of HAMD‐17 total score				
<25	45 (54.9)	48 (56.5)	47 (57.3)	140 (56.2)
≥25	37 (45.1)	37 (43.5)	35 (42.7)	109 (43.8)
Mean (SD)	24.5 (2.1)	24.8 (2.4)	24.6 (2.2)	24.6 (2.2)
Classification of MDD episode based on DSM‐5				
Single episode	36 (43.9)	32 (37.6)	38 (46.3)	106 (42.6)
Recurrent	46 (56.1)	53 (62.4)	44 (53.7)	143 (57.4)
Episode recurrences				
First time	36 (43.9)	32 (37.6)	38 (46.3)	106 (42.6)
Second time	24 (29.3)	30 (35.3)	23 (28.0)	77 (30.9)
Third to seventh time	20 (24.4)	23 (27.1)	18 (22.0)	61 (24.5)
No Less than eight times	1 (1.2)	0	2 (2.4)	3 (1.2)
Unknown	1 (1.2)	0	1 (1.2)	2 (0.8)
Duration of current episode at randomization^†^				
2–4 months	20 (24.4)	30 (35.3)	16 (19.5)	66 (26.5)
4–6 months	19 (23.2)	17 (20.0)	24 (29.3)	60 (24.1)
6–8 months	16 (19.5)	15 (17.6)	17 (20.7)	48 (19.3)
8–10 months	13 (15.9)	11 (12.9)	12 (14.6)	36 (14.5)
10–12 months	11 (13.4)	10 (11.8)	8 (9.8)	29 (11.6)
>12 months	3 (3.7)	2 (2.4)	5 (6.1)	10 (4.0)

*Note*: All data are in *n* (%) unless specified.

Abbreviations: BMI, body mass index; DSM‐5, Diagnostic and Statistical Manual of Mental Disorders, 5th edition; FAS, full analysis set; HAMD‐17, 17‐item Hamilton Rating Scale for Depression; MDD, major depressive disorder; SD, standard deviation.

^†^ Duration of current episode at randomization = (Date of the randomization) − (Onset date) + 1.

### Efficacy

#### Primary endpoint

**Fig. 3:**
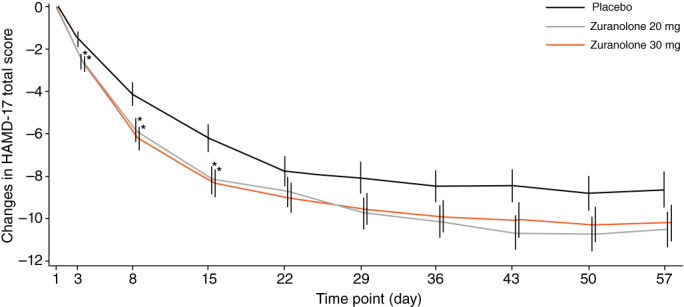
LS mean (SE) change from baseline in HAMD‐17 total score over 57 days (FAS) in the zuranolone 20‐mg and 30‐mg groups compared with placebo. *P < 0.05. FAS, full analysis set; LS, least squares; SE, standard error.

The adjusted mean (standard error [SE]) change from baseline in the HAMD‐17 total score on Day 15 was −6.22 (0.62) in the placebo group, −8.14 (0.62) in the zuranolone 20‐mg group, and − 8.31 (0.63) in the zuranolone 30‐mg group (Table 2 and Fig. 3).

**Table 2:** Analysis of change from baseline in HAMD‐17 total score by time point over 57 days (FAS)

		Observed value			*Vs* placebo	
Time point (planned day)	Treatment group	*n*	Mean (SD)	Change from baseline LS mean (SE)	Difference of LS mean [95% CI]	*P*
1	Placebo	82	24.46 (2.13)	—		
	Zuranolone 20 mg	85	24.85 (2.36)	—	—	—
	Zuranolone 30 mg	82	24.61 (2.20)	—	—	—
3	Placebo	81	22.86 (3.42)	−1.54 (0.36)		
	Zuranolone 20 mg	84	22.14 (3.54)	−2.56 (0.36)	−1.02 [−2.02, −0.03]	0.0442
	Zuranolone 30 mg	82	21.85 (4.23)	−2.72 (0.36)	−1.18 [−2.18, −0.18]	0.0213
8	Placebo	82	20.32 (4.47)	−4.13 (0.52)		
	Zuranolone 20 mg	84	18.93 (4.87)	−5.82 (0.51)	−1.69 [−3.12, −0.26]	0.0206
	Zuranolone 30 mg	81	18.32 (5.60)	−6.21 (0.52)	−2.08 [−3.52, −0.63]	0.0049
15	Placebo	82	18.23 (5.43)	−6.22 (0.62)		
	Zuranolone 20 mg	81	16.52 (6.07)	−8.14 (0.62)	−1.92 [−3.65, −0.19]	0.0296
	Zuranolone 30 mg	80	16.16 (6.11)	−8.31 (0.63)	−2.09 [−3.83, −0.35]	0.0190
22	Placebo	79	16.66 (6.78)	−7.76 (0.70)		
	Zuranolone 20 mg	81	15.74 (6.31)	−8.74 (0.69)	−0.98 [−2.92, 0.95]	0.3173
	Zuranolone 30 mg	77	15.29 (6.33)	−9.01 (0.70)	−1.25 [−3.20, 0.70]	0.2080
29	Placebo	79	16.27 (7.03)	−8.09 (0.73)		
	Zuranolone 20 mg	75	14.25 (6.36)	−9.72 (0.73)	−1.63 [−3.66, 0.41]	0.1167
	Zuranolone 30 mg	76	14.86 (6.54)	−9.55 (0.74)	−1.46 [−3.51, 0.59]	0.1632
36	Placebo	78	15.82 (7.06)	−8.46 (0.77)		
	Zuranolone 20 mg	75	13.75 (6.56)	−10.14 (0.77)	−1.67 [−3.81, 0.47]	0.1249
	Zuranolone 30 mg	74	14.28 (7.27)	−9.89 (0.78)	−1.43 [−3.59, 0.72]	0.1922
43	Placebo	74	15.59 (7.32)	−8.46 (0.80)		
	Zuranolone 20 mg	75	13.24 (7.18)	−10.67 (0.80)	−2.22 [−4.44, 0.01]	0.0509
	Zuranolone 30 mg	71	13.92 (7.30)	−10.05 (0.81)	−1.60 [−3.84, 0.64]	0.1614
50	Placebo	74	15.42 (7.34)	−8.79 (0.81)		
	Zuranolone 20 mg	72	12.86 (6.89)	−10.73 (0.82)	−1.94 [−4.21, 0.33]	0.0938
	Zuranolone 30 mg	72	13.60 (7.61)	−10.29 (0.83)	−1.50 [−3.78, 0.79]	0.1974
57	Placebo	71	15.24 (7.50)	−8.63 (0.83)		
	Zuranolone 20 mg	70	13.16 (6.92)	−10.52 (0.83)	−1.89 [−4.20, 0.43]	0.1094
	Zuranolone 30 mg	70	13.76 (7.67)	−10.17 (0.84)	−1.54 [−3.87, 0.78]	0.1922

*Note*: Fixed effect: treatment group, time point, interaction effect (treatment group and time point).Covariate: baseline value of HAMD‐17 total score, sex (male, female).Covariance structure: unstructured .Effect size is defined as the difference of LS mean divided by common SD.

Abbreviations: CI, confidence interval; FAS, full analysis set; HAMD‐17, 17‐item Hamilton Rating Scale for Depression; LS, least square; SD, standard deviation; SE, standard error.

The difference in the adjusted mean between the zuranolone 30‐mg and placebo groups (−2.09; 95% confidence interval [CI]: −3.83, −0.35; *P* = 0.0190) and between the zuranolone 20‐mg and placebo groups (−1.92; 95% CI: −3.65, −0.19; *P* = 0.0296) was statistically significant on Day 15.

Nominally significant improvement (*P* < 0.05) in the HAMD‐17 total score was observed in the zuranolone 20‐ and 30‐mg groups when compared with the placebo group during the early treatment period on Days 3 and 8 (Table 2 and Fig. 3). Thereafter, the difference in the HAMD‐17 total score between the groups was not significant up to Day 57. (*P* ≥ 0.05; Table 2 and Fig. 3).

#### Secondary endpoints

**Fig. 4:**
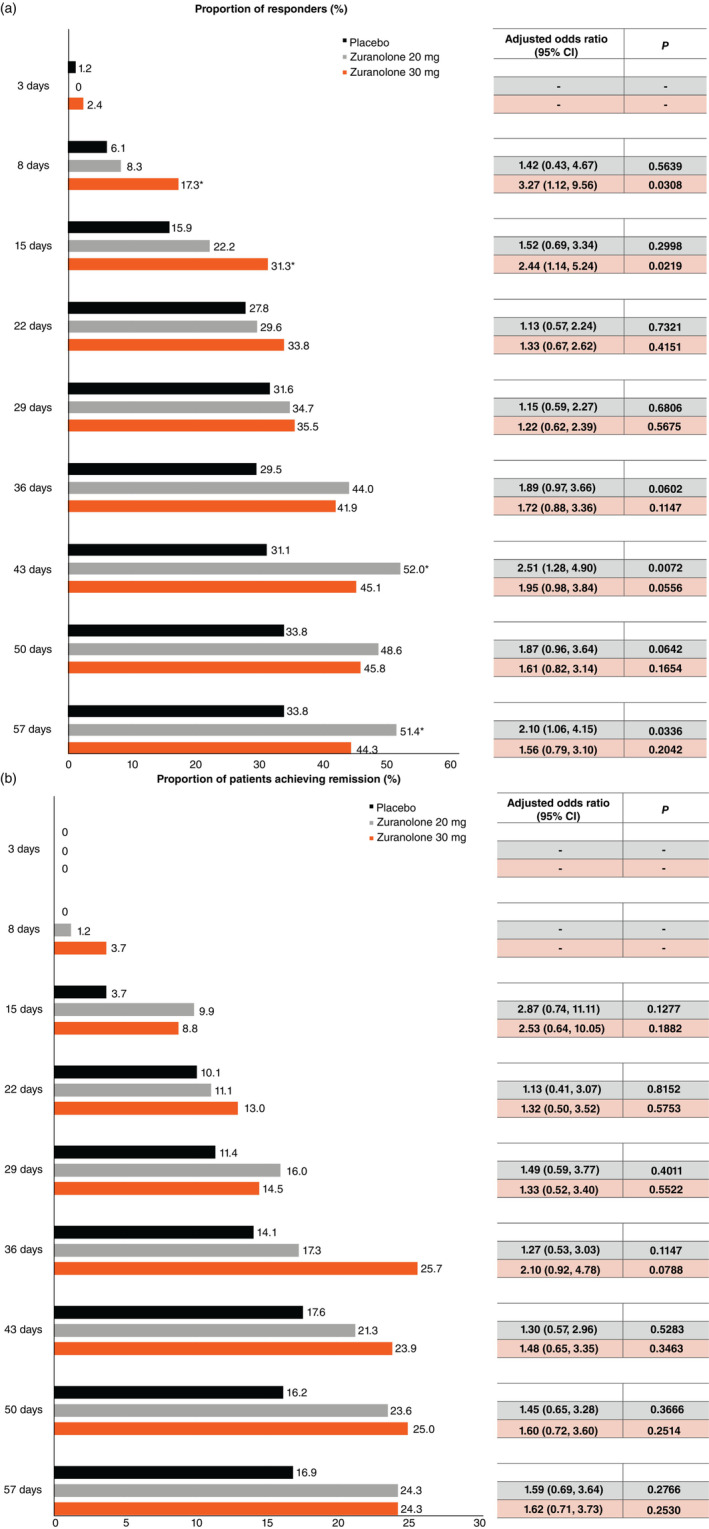
Bar graph of (a) response and (b) remission by HAMD‐17 score over 57 days (FAS). *P < 0.05. IPW‐GEE analysis. The data from Day 3 were excluded from the model so that the model could be converged, and ‘‐’ is shown at the timepoint. Fixed effect: treatment group, time point, interaction effect (treatment group and time point). Covariate: baseline value of HAMD‐17 total score, sex (male, female). Working correlation structure: independent. Response: Number of patients with nonmissing HAMD‐17 total score at the visit as well as at baseline were evaluated. Number of patients with a response were defined as a reduction of ≥50% in the baseline HAMD‐17 total score. The denominator of the percentage is the number of patients with nonmissing HAMD‐17 total score at the visit as well as at baseline. Remission: Number of patients with nonmissing HAMD‐17 total score at the visit were evaluated. Number of patients with a remission were defined as a score of ≤7 in the HAMD‐17. The denominator of the percentage is the number of patients with nonmissing HAMD‐17 total score at the visit. CI, confidence interval; FAS, full analysis set; HAMD‐17, 17‐item Hamilton Rating Scale for Depression; IPW‐GEE, inverse probability‐weighted generalized estimating equation.

The response rate (≥50% reduction from the baseline HAMD‐17 total score) on Days 8 and 15 was higher in the zuranolone 20‐ and 30‐mg groups than in the placebo group. The adjusted odds ratio (OR) for responding to treatment on Day 15 between the zuranolone 30‐mg and the placebo groups was 2.44 (95% CI: 1.14, 5.24), with a nominally significant difference (*P* < 0.05) in favor of the zuranolone 30‐mg group (Fig. 4a). A trend of sustained higher odds of response rate in the zuranolone groups when compared with the placebo group was observed during follow‐up period A (up to Day 57); however, the differences were not nominally significant between the groups (*P* ≥ 0.05), and nominally significant difference (*P* < 0.05) were seen on Day 43 and Day 57 between the zuranolone 20 mg and placebo groups (Fig. 4a).

Compared with placebo, both zuranolone groups had higher remission rates on Day 15 (adjusted OR: zuranolone 20 mg vs placebo, 2.87 [95% CI: 0.74, 11.11]; zuranolone 30 mg vs placebo, 2.53 [95% CI: 0.64, 10.05]); however, the differences were not significant (Fig. 4b). Similarly, compared with placebo, more patients achieved remission in the zuranolone groups during follow‐up period A; however, the differences in the OR for remission were not significantly different between the groups (Fig. 4b).

The mean improvements in HAMD‐17 core, anxiety, Bech‐6, and Maier scores on Day 15 were numerically greater in the zuranolone 20‐ and 30‐mg groups than in the placebo group, but the differences in least‐squares (LS) mean were not nominally significantly different (Table 3). For the HAMD‐17 core score, the differences in the LS mean were − 2.56 (95% CI: −6.53, 1.40; *P* = 0.2041) between the zuranolone 20‐mg and placebo groups and − 2.84 (95% CI: −6.84, 1.16; *P* = 0.1635) between the zuranolone 30‐mg and placebo groups. For the HAMD‐17 anxiety score, the differences in the LS mean were − 2.89 (95% CI: −6.58, 0.80; *P* = 0.1240) between the zuranolone 20‐mg and placebo groups and − 1.73 (95% CI: −5.44, 1.99; *P* = 0.3611) between the zuranolone 30‐mg and placebo groups. However, for the HAMD‐17 insomnia symptoms subscale score, nominally significant differences (*P* < 0.05) were observed in the zuranolone 20‐ and 30‐mg groups, when compared with the placebo group, on Day 15 (Table 3) but not during follow‐up period A (data not shown).

**Table 3:** Analysis of change from baseline in HAMD‐17 subscale scores by timepoint (FAS)

		Baseline observed value		Day 15 observed value			*Vs* placebo	
Subscale	Treatment group	*n*	Mean (SD)	*n*	Mean (SD)	Change from baseline LS mean (SE)	Difference of LS mean [95% CI]	*P*
Core	Placebo	82	47.13 (6.94)	82	34.76 (12.88)	−12.39 (1.43)	—	—
	Zuranolone 20 mg	85	48.35 (7.84)	81	33.15 (14.80)	−14.95 (1.42)	−2.56 [−6.53, 1.40]	0.2041
	Zuranolone 30 mg	82	46.95 (8.23)	80	31.56 (14.22)	−15.22 (1.44)	−2.84 [−6.84, 1.16]	0.1635
Anxiety	Placebo	82	43.77 (8.07)	82	32.52 (11.85)	−11.20 (1.33)	—	—
	Zuranolone 20 mg	85	45.10 (7.34)	81	30.32 (13.27)	−14.09 (1.32)	−2.89 [−6.58, 0.80]	0.1240
	Zuranolone 30 mg	82	43.77 (9.25)	80	30.69 (14.33)	−12.92 (1.34)	−1.73 [−5.44, 1.99]	0.3611
Bech‐6	Placebo	82	58.76 (7.59)	82	43.13 (14.20)	−15.54 (1.68)		
	Zuranolone 20 mg	85	59.89 (7.69)	81	41.58 (16.72)	−17.72 (1.67)	−2.18 [−6.83, 2.48]	0.3583
	Zuranolone 30 mg	82	57.10 (8.27)	80	39.72 (15.71)	−17.31 (1.70)	−1.76 [−6.46, 2.94]	0.4608
Maier	Placebo	82	51.58 (8.53)	82	37.04 (14.10)	−14.40 (1.52)		
	Zuranolone 20 mg	85	52.11 (7.65)	81	35.34 (14.42)	−16.22 (1.50)	−1.82 [−6.01, 2.38]	0.3944
	Zuranolone 30 mg	82	49.75 (7.81)	80	33.44 (14.39)	−16.25 (1.53)	−1.85 [−6.09, 2.39]	0.3910
Insomnia symptoms	Placebo	82	69.31 (21.19)	82	50.61 (28.86)	−18.96 (3.05)		
	Zuranolone 20 mg	85	70.00 (19.55)	81	40.12 (29.90)	−29.73 (3.02)	−10.77 [−19.21, −2.34]	0.0125
	Zuranolone 30 mg	82	74.19 (19.80)	80	37.50 (29.83)	−34.86 (3.08)	−15.91 [−24.43, −7.38]	0.0003

*Note*: The difference between individual scores in the core, anxiety, Bech‐6, and Maier subscale items was not significant (data not shown in the table).Fixed effect: treatment group, time point, interaction effect (treatment group and time point).Covariate: baseline value of each HAMD‐17 subscale score, sex (male, female).Covariance structure: core (unstructured), anxiety (unstructured), Bech‐6 (unstructured), Maier (unstructured), insomnia symptoms (unstructured).

Abbreviations: CI, confidence interval; FAS, full analysis set; HAMD‐17, 17‐item Hamilton Rating Scale for Depression; LS, least square; SD, standard deviation; SE, standard error.

Over 57 days, no difference could be demonstrated between the placebo and zuranolone 20‐ and 30‐mg groups using assessments such as CGI‐S (‘normal, not at all ill’ or ‘borderline mentally ill’; Table S3), PGI‐I (‘very much better’ or ‘much better’), PHQ‐9, and change from baseline in SF‐36 summary scores or 8‐domain scores.

Nominally significant differences (*P* < 0.05) showing improvement in the ISI total scores were observed with zuranolone 30 mg versus placebo as early as Day 3. The reduction in the ISI total score on Day 15 was greater in the zuranolone 20‐ and 30‐mg groups than in the placebo group, with nominally significant differences (*P* < 0.05) when compared with the placebo group. No significant differences were observed in the ISI total score between the zuranolone 20‐ or 30‐mg group and the placebo group during follow‐up period A (Table S4).

#### Safety

Patients in this study received the study drug for a mean (SD) and median (min, max) of 13.9 (0.4) days and 14 (12, 14) days, respectively, in the placebo group, 13.5 (1.7) days and 14 (3, 14) days, respectively, in the zuranolone 20‐mg group, and 13.5 (1.7) days and 14 (2, 14) days, respectively, in the zuranolone 30‐mg group.

No deaths or other serious TEAEs were reported during the study period (Table 4). TEAEs were reported in a similar proportion of patients in the placebo (52.4%), zuranolone 20‐mg (54.1%), and 30‐mg groups (54.9%) (Table 4).

**Table 4:** Overall summary of incidence of TEAEs during the study period (safety population)

	Placebo *N* = 82 *n* (%)	Zuranolone 20 mg *N* = 85 *n* (%)	Zuranolone 30 mg *N* = 82 *n* (%)
TEAEs			
n (%)	43 (52.4)	46 (54.1)	45 (54.9)
Number of events	84	90	109
TEAEs ≥5%			
Infections and infestations	7 (8.5)	9 (10.6)	12 (14.6)
Nasopharyngitis	4 (4.9)	6 (7.1)	7 (8.5)
Nervous system disorders	15 (18.3)	23 (27.1)	26 (31.7)
Somnolence	5 (6.1)	10 (11.8)	17 (20.7)
Dizziness	4 (4.9)	9 (10.6)	8 (9.8)
Headache	3 (3.7)	6 (7.1)	5 (6.1)
Gastrointestinal disorders	14 (17.1)	13 (15.3)	11 (13.4)
Musculoskeletal and connective tissue disorders	6 (7.3)	4 (4.7)	2 (2.4)
General disorders and administration site conditions	7 (8.5)	1 (1.2)	8 (9.8)
Investigations^†^	12 (14.6)	9 (10.6)	11 (13.4)
TEAEs leading to death			
*n* (%)	0 (0)	0 (0)	0 (0)
Serious TEAEs other than death			
*n* (%)	0 (0)	0 (0)	0 (0)
TEAEs leading to withdrawal of study drug			
*n* (%)	0 (0)	1 (1.2)	0 (0)
Number of events	0	2	0
TEAEs of special interest			
*n* (%)	9 (11.0)	18 (21.2)	22 (26.8)
Number of events	10	21	25
Sedation	0	1 (1.2)	0
Somnolence	5 (6.1)	10 (11.8)	17 (20.7)
Dizziness	4 (4.9)	9 (10.6)	8 (9.8)
Respiratory failure (SMQ)	0	0	0
Drug abuse and dependence (SMQ)	0	0	0

*Note*: ‘During the study’ denotes the time window from the start of the treatment period to the end of the follow‐up period B. TEAEs of special interest were defined as all events related to sedation, somnolence, dizziness, respiratory failure, and suspected to be drug dependence (events classified as drug abuse and dependence in the International Council for Harmonization of Technical Requirements for Pharmaceuticals for Human Use Medical Dictionary for Regulatory Activities [MedDRA], by the Standardized [MedDRA Queries]). AEs were coded using MedDRA Version 23.0 update. AE, adverse event; MedDRA, Medical Dictionary for Regulatory Activities; SMQ, Standardized MedDRA Queries; TEAE, treatment‐emergent adverse event.

^†^ Investigations include assessments related to blood, urine, respiratory system, electrocardiogram, and patient weight.

Treatment‐related AEs were reported in a greater proportion of patients in the zuranolone 20‐mg (31.8%) and 30‐mg groups (32.9%) than in the placebo group (20.7%). Of the TEAEs, treatment‐related AEs of the nervous system included somnolence (placebo [3.7%], zuranolone 20 mg [10.6%], zuranolone 30 mg [20.7%]), dizziness (3.7%, 9.4%, 9.8%, respectively), and headache (3.7%, 3.5%, 1.2%, respectively) (Table S5).

The study drug was withdrawn in one patient (1.2%) from the zuranolone 20‐mg group due to two TEAEs (dizziness and somnolence; Table 4). Dizziness was of moderate severity, and somnolence was of mild severity; both events resolved over time and were considered treatment‐related AEs.

The incidence of TEAEs of special interest during the study period was higher in the zuranolone 20‐mg (21.2%) and 30‐mg groups (26.8%) than in the placebo group (11.0%). The most common TEAE of special interest was somnolence (placebo, 6.1%; zuranolone 20 mg, 11.8%; zuranolone 30 mg, 20.7%), followed by dizziness (placebo, 4.9%; zuranolone 20 mg, 10.6%; zuranolone 30 mg, 9.8%; Table 4). No TEAEs classified as respiratory failure or drug abuse and drug dependence were reported. Most TEAEs of special interest were considered treatment related. No withdrawal symptoms were reported during the study period (Table S6).

No clinically notable trends were observed in the laboratory values, vital signs, or electrocardiographic parameters between the groups during the study period (data not shown).

The Dependence Assessment Committee found no events suspected to be related to drug dependence or drug abuse. Based on spirometry data, zuranolone did not show any apparent effects on respiratory function (data not shown).

#### Pharmacokinetics

**Fig. 5:**
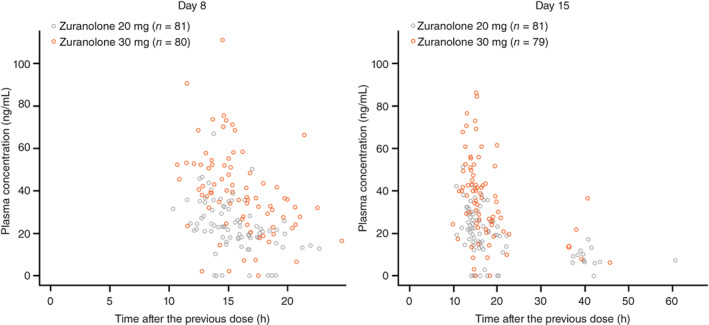
Scatter plot of plasma concentration (ng/mL) of zuranolone on Day 8 (visit 3) and Day 15 (visit 4).

Plasma zuranolone concentrations were dose dependent and tended to be higher in the zuranolone 30‐mg than in the 20‐mg group. Specifically, plasma zuranolone concentrations in both the zuranolone 20‐ and 30‐mg groups were comparable (steady state) between Days 8 (visit 3) and 15 (visit 4) (Fig. 5).

CONSORT checklist is presented as Table S7.

## Discussion

This multicenter, randomized, double‐blind, placebo‐controlled, parallel‐group study was conducted in Japanese patients with MDD to evaluate the safety and efficacy of zuranolone 20‐ and 30‐mg once‐daily oral administration for 14 days in comparison with placebo.

Dose selection was based on the safety and tolerability outcome of the 30‐mg dose from the previous phase 1 study conducted in Japanese healthy adults, including elderly adults; similar plasma exposure levels of zuranolone were observed between Japanese and White healthy adults (Sonoyama *et al*., article under submission). Improvement in depressive symptoms with zuranolone 30 mg once daily for 14 days with no major safety and tolerability issues was observed in the phase 2 study conducted in patients with MDD in the US.^31^

In this study, the primary efficacy endpoint of change from baseline in the HAMD‐17 total score on Day 15 was met; the mean (SD) HAMD‐17 total score improved significantly from Day 3 (first observation) to Day 15 (day after last dose) with zuranolone 20‐mg and 30‐mg doses versus placebo. Thereafter, despite a numerical difference in HAMD‐17 total score, there was no statistical difference between the groups during the follow‐up period. These results were also reflected in the response rate and remission rate that were higher in the zuranolone groups versus placebo group over 15 days, with a nominally significant difference observed for the response rate on Days 8 and 15 in the zuranolone 30‐mg group versus placebo group. These results showed a rapid treatment onset and effect of zuranolone as an antidepressant. We observed that the higher numerical response rate and remission rate in the zuranolone 20‐ and 30‐mg groups compared with the placebo group were sustained in the follow‐up period; however, the differences were not nominally significant.

Additionally, the HAMD‐17 insomnia symptom score showed nominally significant differences in the zuranolone 20‐ and 30‐mg groups when compared with the placebo group at Day 15. Of note, three patients in the placebo group failed to meet the scheduled visits specified in the protocol due to the coronavirus disease (COVID‐19) pandemic. While these noncompliances occurred during follow‐up period A or B, they had no meaningful impact on the assessments.

The results of this study were different from those of the previous randomized, double‐blind, placebo‐controlled, phase 2 study conducted in the US, which reported a larger LS mean (±SD) change versus this study in the HAMD‐17 score from baseline on Day 15 between the zuranolone 30‐mg once‐daily group (−17.4 [1.3] vs −8.31 [0.63]) and the placebo group (−10.3 [1.3] vs −6.22 [0.62]; LS mean difference in change: [−7.0; 95% CI: −10.2, −3.9; *P* < 0.001] vs [−2.09; 95% CI: −3.83, −0.35; *P* = 0.0190]). These differences could be attributed to differences in patient demographics and characteristics, race, baseline HAMD‐17 scores (mean [SD]: zuranolone, 25.2 [2.6]; placebo, 25.7 [2.4] in the US study), a smaller sample size in the US study, and differences in the study design (the US study admitted patients as being inpatients for the first week although they were enrolled from outpatient clinics).^31^ The pivotal, double‐blind, randomized, placebo‐controlled, phase 3 WATERFALL study evaluated the efficacy and safety of zuranolone 50 mg in adults aged 18–64 years with MDD (*n* = 543). The primary endpoint was met with zuranolone 50 mg, with an improvement in depressive symptoms with zuranolone 50 mg once daily (change from baseline in LS means [SE]: −14.1 [0.51]) and placebo (−12.3 [0.50]) and LS mean difference (−1.7 points; *P* = 0.0141) on Day 15, as assessed using the HAMD‐17 total score.^29^

With regard to rapid onset antidepressants, intranasal esketamine, a noncompetitive *N*‐methyl *D*‐aspartate receptor antagonist, is indicated in combination with an oral antidepressant for both ‘TRD’ and ‘MDD with acute suicidal ideation or behavior’ in adults. The recommended dosage of intranasal esketamine for the latter is 84 mg twice per week for 4 weeks; it may be reduced to 56 mg twice per week based on tolerability. After 4 weeks, evidence of therapeutic benefit should determine the need for continued treatment.^35^ The double‐blind, phase 3 ASPIRE I and ASPIRE II studies in MDD patients with active suicidal ideation showed greater improvement in the Montgomery‐Asberg Depression Rating Scale total score with intranasal esketamine (twice weekly for 4 weeks) + standard‐of‐care vs placebo + standard‐of‐care at 24 h (LS mean difference [SE]: −3.82 [1.39]; 95% CI: −6.56, −1.09; −3.9 [1.39], 95% CI: −6.60, 1.11; *P* = 0.006 for both, respectively) and thereafter over the 4‐week double‐blind treatment.^36^, ^37^ Although a direct comparison cannot be made, the improvement in the zuranolone 20‐ and 30‐mg groups vs the placebo group was similar to that achieved with rapid‐acting agents that exert their effects *via* modulation of glutamate/GABA neurotransmission and impact excitatory‐inhibitory balance in brain networks^36^, ^37^ and better than that with other antidepressants.^38^ Important evidence for the antidepressant mechanism hypothesis of zuranolone includes presence of decreased allopregnanolone in patients with major depression,^22^ abundant distribution of allopregnanolone in the amygdala,^25^ and an antidepressant effect mediated by enhancement of amygdala θ activity due to enhancement of extrasynaptic GABA receptor function on inhibitory neurons in the amygdala.^26^ Benzodiazepine drugs, which are synaptic GABAA receptor potentiators and are indicated for anti‐anxiety and sleep effects, do not enhance amygdala θ activity.^26^ Based on the above, Zuranolone is thought to exert a rapid antidepressant effect by compensating for the weakened GABA enhancement effect of allopregnanolone.

Overall, this study showed rapid onset of action for zuranolone, compared with the apparent results of the meta‐analysis of antidepressants published by Posternak and Zimmerman in 2005 and other studies analyzing the time of action onset for SSRIs, SNRIs, mirtazapine, and vortioxetine that are frequently used as first‐line therapy. A meta‐analysis of 76 double‐blind, placebo‐controlled trials shows that in general, the effect of antidepressant is slow.^6^, ^39^ Similarly, another meta‐analysis of 28 placebo‐controlled, randomized controlled trials (*n* = 5872) of SSRIs reported that the treatment response was greatest in the first week.^7^ Evidence also suggests that response can be observed by Week 2 with fluoxetine,^8^ mirtazapine, venlafaxine, SSRI augmentation with pindolol,^9^ and vortioxetine.^10^ However, direct comparisons are difficult due to differences in trial design.^40^ Nevertheless, these data provide an estimate of when to expect drug‐placebo separation as a marker of treatment response.

The Incidence of TEAEs was comparable across treatment groups. All TEAEs were mild or moderate in severity, and no deaths or serious TEAEs were reported during the study period. The incidence of TEAEs of special interest was higher in the zuranolone groups than in the placebo group. Of the TEAEs of special interest, somnolence appeared to be dose dependent: 11.8% in the zuranolone 20‐mg group, 20.7% in the zuranolone 30‐mg group, and 6.1% in the placebo group. However, somnolence was an expected event resulting from the mechanism of action of zuranolone and this observation was consistent with the safety profile reported in previous studies.^29^, ^31^

In this study, plasma zuranolone concentrations were dose dependent, which aligns with the findings from the phase 1 study conducted in Japanese healthy adults (Sonoyama *et al*., article under submission).

The results should be compared and interpreted with caution, considering the small sample size and short duration of this study. Additionally, patients with definite suicidal ideation were excluded from the study, and thus this drug cannot be evaluated for suicidal ideation. However, it can be inferred that the rapid antidepressant effect may also be useful in the treatment of suicidal ideation. Further, the effect of concomitant use with antidepressants cannot be evaluated from this study and is being evaluated in an ongoing phase 3 study.

## Conclusion

This phase 2 study demonstrated the efficacy of oral zuranolone 20 and 30 mg administered once daily for 14 days in Japanese patients with MDD, and no new safety issues were identified. The encouraging results of this study are being confirmed in an ongoing phase 3 study.

## Author contributions

M.K., K.N., T.I., and T.M: conception and design, interpretation of results, and review of the manuscript. T.S.: analysis of data and review of the manuscript. D.O. and H.Y.: conception and design, acquisition and analysis of data, and review of the manuscript. T.B. and R.S.: conception and design, analysis of data, and review of the manuscript. T.M.: conception and design, analysis of data, interpretation of results, and review of the manuscript.

## Disclosure statement

M.K. received support for the present manuscript (e.g. funding, provision of study materials, medical writing, article processing charges, etc.) from Shionogi & Co., Ltd.; consulting fees from Sumitomo Pharma Co., Ltd., Otsuka Pharmaceutical Co., Lundbeck Japan, Takeda Pharmaceutical Co., and Shionogi & Co., Ltd. in the past 36 months; and payment/honoraria from Sumitomo Pharma Co., Ltd., Otsuka Pharmaceutical Co., Meiji‐Seika Pharma Co., Ltd., Eli Lilly, MSD K.K., Pfizer, Janssen Pharmaceutical, Shionogi & Co., Ltd., Mitsubishi Tanabe Pharma Corp., Takeda Pharmaceutical Co., Ltd., Lundbeck Japan, Viatris, Eisai, Kyowa Pharmaceutical Inc., and Ono Pharmaceutical in the past 36 months. K.N. received grants paid to his institution from Shionogi & Co., Ltd., Sumitomo Pharma Co., Ltd., Otsuka Pharmaceutical Co., Meiji‐Seika Pharma Co., Ltd., Janssen Pharmaceutical, Mitsubishi Tanabe Pharma Corp., Nippon Boehringer Ingelheim Co., Ltd., and Mochida Pharmaceutical Co., Ltd. in the past 36 months; payment/honoraria from Sumitomo Pharma Co., Ltd., Otsuka Pharmaceutical Co., Meiji‐Seika Pharma Co., Ltd., Janssen Pharmaceutical, Mitsubishi Tanabe Pharma Corp., Takeda Pharmaceutical Co., Ltd., Lundbeck Japan, Viatris, Eisai Co., Ltd., Nippon Boehringer Ingelheim Co., Ltd., Mochida Pharmaceutical Co., Ltd. in the past 36 months; and support for attending meetings and/or travel from Sumitomo Pharma Co., Ltd., Otsuka Pharmaceutical Co., Meiji‐Seika Pharma Co., Ltd., Janssen Pharmaceutical, Mitsubishi Tanabe Pharma Corp., Takeda Pharmaceutical Co., Ltd., Nippon Boehringer Ingelheim Co., Ltd., Mochida Pharmaceutical Co., Ltd. in the past 36 months. T.I. received grants or contracts from Shionogi & Co., Ltd., Daiichi Sankyo Inc., Eisai Co., Ltd., Otsuka Pharmaceutical, Co., Ltd., Sumitomo Pharma Co., Ltd., and Tsumura in the past 36 months; consulting fees from Shionogi, Takeda Pharmaceutical Co., Ltd., Viatris, and Ono Pharmaceutical in the past 36 months; payment/honoraria from Otsuka Pharmaceutical, Yoshitomiyakuhin, Shionogi, Mitsubishi Tanabe Pharma Corp, Takeda Pharmaceutical Co., Ltd., Kyowa Pharmaceutical Industry, Mochida Pharmaceutical, Pfizer, MSD K.K., Meiji‐Seika Pharma, Eisai, Novartis Pharma, Sumitomo Pharma Co., Ltd., Taisho Toyama Pharmaceutical, Tsumura, and Janssen Pharmaceutical. T.S., T.B., and R.S. are full‐time employees and own stocks *via* employee stock ownership society of Shionogi & Co., Ltd. D.O. was a full‐time employee during the study period and owns stocks of Shionogi & Co., Ltd. H.Y. and T.M. are full‐time employees of Shionogi & Co., Ltd.

## Supporting information


**Table S1.** Inclusion criteria, exclusion criteria, and statistical analysis.
**Table S2.** Discontinuation of study drug and patient discontinuation/withdrawal criteria.
**Table S3.** Analysis of CGI‐S by timepoint (FAS).
**Table S4.** Changes from baseline in ISI total score by timepoint (FAS).
**Table S5.** Incidence of treatment‐related AEs during the study period (safety population).
**Table S6.** Incidence of treatment‐related AEs of special interest during the study period (safety population).

**Table S7.** CONSORT checklist.

## Data Availability

Shionogi & Co., Ltd., is committed to disclosing the synopses and results of its clinical trials and sharing clinical trial data (raw dataset or study data tabulation model dataset) with researchers upon reasonable request. The data will be available when the medicine and the indication have received the regulatory approval in JP, US, and EU on 5 December 2018, or later, and the primary manuscript describing the results has been accepted for publication. If the research proposal is reviewed by an independent review panel and approved, the anonymized data and redacted documents will be provided in a secure research environment. For further details, please refer to the websites of Shionogi & Co., Ltd. (https://www.shionogi.com/shionogi/global/en/company/policies/shionogi‐group‐clinical‐trial‐data‐transparency‐policy.html) and Vivli (https://vivli.org/).
