# Supplementary material for: Efficacy and safety of zuranolone in Japanese adults with major depressive disorder: A double‐blind, randomized, placebo‐controlled, phase 2 clinical trial
Source: Psychiatry Clin Neurosci. 2023 Jun 21;77(9):497–509. doi: 10.1111/pcn.13569 (PMC11488630; doi:10.1111/pcn.13569)
Supplement: Supplementary file 1 — Table S1. Inclusion criteria, exclusion criteria, and statistical analysis. Table S2. Discontinuation of study drug and patient discontinuation/withdrawal criteria. Table S3. Analysis of CGI‐S by timepoint (FAS). Table S4. Changes from baseline in ISI total score by timepoint (FAS). Table S5. Incidence of treatment‐related AEs during the study period (safety population). Table S6. Incidence of treatment‐related AEs of special interest during the study period (safety population). [file PCN-77-497-s001.docx]

**SUPPLEMENTARY MATERIAL**

**Table S1.** Inclusion criteria, exclusion criteria, and statistical analysis

| **Inclusion criteria**   - Japanese male or female outpatients aged between ≥18 years and ≤75 years at the time of signing the ICF; voluntary written informed consent was provided by the patients or their legally acceptable representatives (for patients aged <20 years). - Patients who were evaluated using M.I.N.I. and diagnosed as having MDD according to the DSM-5 and who met the following two conditions:   - The current MDD episode was ongoing for at least 8 weeks before the day of signing the ICF.   - Duration of the current MDD episode was ≤12 months before signing the ICF. - Patients with a HAMD-17 total score of ≥22 and a PHQ-9 total score of ≥15 at visit 1. - Patients with FEV1 of ≥70% and percent vital capacity by respiratory function test (spirometry) of ≥80%. - Patients who agreed to refrain from alcohol from 7 days prior to visit 1 to 7 days after the end of study drug administration. - Patients who were able to maintain the daily rhythm of eating an evening meal or a meal before bedtime and sleeping at night from the time of signing the ICF until the completion of the study. - Male patients were deemed eligible if they followed the following rule during the treatment period and for at least 26 days after the last dose of the study drug:   - Abstained from sperm donation.   And agreed to any of the following:   - Remained heterosexually or homosexually abstinent as a preferred usual lifestyle of patients (no sexual intercourses for a long period of time).   or   - Used the following methods of contraception:   - In case of sexual intercourse with women not currently pregnant but of childbearing potential, male condoms had to be used. Further, since male condoms may be damaged and result in leakage, patients had to be informed of the fact that effective methods of contraception was beneficial for female partners.   - Male condoms had to be used in any activity that might expose others to semen.   - If the female partner of a male participant was not currently pregnant but of childbearing potential, the female partner was to also use appropriate methods of contraception. - Female patients were deemed eligible if they were not pregnant or breastfeeding and at least met any of the following criteria:   - Not of childbearing potential   or   - - Women of childbearing potential who used the method of contraception, preferably less user dependent and extremely effective with a failure rate of below 1% per year, during the treatment period and for at least 26 days after the last dose of the study drug and agreed to abstain from egg donation for reproduction (egg, oocytes) during the treatment period and for at least 26 days after the last dose of the study drug. The investigator or subinvestigator was to determine the eligibility considering the relationship between the effectiveness of the method of contraception and   the initial dose of the study drug.   - - Women of childbearing potential had to undergo highly sensitive pregnancy test (urine test) within 24 hours prior to the initial dose of the study drug and had to test negative.   The pregnancy tests were specified during the treatment period and additionally required after the study treatment.  The investigator or subinvestigator was responsible to confirm the medical history, menstrual history, and latest status of sexual activity in order to reduce the risk of enrolling women whose pregnancy was in its early stage and had  not been detected.  **Additional exclusion criteria**   - Patients with TRD (no improvement in depressive symptoms with the use of ≥2 different antidepressants [excluding antipsychotics] at adequate doses approved in Japan for four weeks for the existing depressive episode; MGH ATRQ was used for the evaluation). - Patients undergoing therapies such as vagal nerve stimulation, electroconvulsive therapy, and transcranial magnetic stimulation for the current depressive episode. - Patients evaluated using M.I.N.I. during the screening period and who had a complication or history of a disease classified into any of the following DSM-5 classifications in the opinion of the investigator or subinvestigator:   - Neurodevelopmental disorders   - Schizophrenia spectrum and other psychotic disorders   - Bipolar and related disorders   - Psychological trauma- and stress-related disorders   - Personality disorders   - Obsessive-compulsive and related disorders   - Anorexia nervosa, bulimia nervosa, binge-eating disorder   - Neurocognitive disorders   - Substance use disorders^†^ - Patients with any of the following diseases:   - Epilepsy (including history of epilepsy)   - Sleep apnea syndrome^‡^   - Chronic obstructive pulmonary disease   - Interstitial pneumonia   - Severe bronchial asthma   - Alveolar hypoventilation syndrome   - Chronic respiratory failure   - Pulmonary hypertension   - Clinically symptomatic bronchiectasis   - Patients with other chronic respiratory diseases and who are deemed ineligible for the study in the opinion of the investigator. - Patients with serious§ hepatic disorder, renal disorder, cardiac disease, pulmonary disease, hematological disease, metabolic disease, etc. - Patients who had gastric bypass surgery, a gastric sleeve, lap band, or any related procedures that interfere with gastrointestinal transit. - Patients with a QTc (QTcF) of >450 msec. - Patients who were considered by the investigator or subinvestigator as having clinical problems with the following results: - 12-lead ECG and medical examination performed at visit pre and visit 1 - Laboratory tests performed at the screening - Patients who deviated from the regulations of prohibited or restricted concomitant medication/therapy (see Table S2) before visit 1. - Patients who tested positive for urine drug test, excluding the cases where the drug detected only at visit pre had been administered undoubtedly for the treatment of depression. - Female patients who tested positive for pregnancy test, who were pregnant, or lactating between visit pre and visit 1, and who wished to become pregnant during the study period, and male patients who wished to impregnate his partner. - Patients who had consumed grapefruit, seville orange, or food or beverages containing these within 14 days prior to visit 1. - Patients who had consumed alcohol-containing food or beverages within seven days prior to visit 1. - Patients who had been administered other investigational drugs within 30 days prior to the visit to the study site. - Patients who had previously participated in clinical trials of zuranolone or SAGE-217. - Patients with known allergy to zuranolone, allopregnanolone, or any related substances. - Patients who were considered to be ineligible for the study by the investigator or subinvestigator, including those who had difficulty in completing the study, who might suffer disadvantages due to participation in the study, for whom the safety could not be assured during the study period, and who were not appropriate for the assessment of the efficacy and safety of the study drug. - Patients at suicidal risk who met any of the following criteria:   - At visit pre and within 12 months prior to visit pre, patients who answered “Yes” to Suicidal Ideation Questions 4 or 5, or any of the Suicidal Behavior Questions (excluding questions about self-injurious behavior without suicidal intent) of the C-SSRS.   - At visit 1, patients who answered “Yes” to Suicidal Ideation Questions 4 or 5 or to any of the Suicidal Behavior Questions (excluding questions about self-injurious behavior without suicidal intent) of the C-SSRS. |
| --- |
| **Sample size**  In this study, a between-group difference of −3.5 and an SD of 7.50 was set, which is within the range (−5.9 to −3.2) of the between-group differences obtained in Part B of the phase 2 and phase 3 studies for MDD conducted in the US, and the effect size was estimated to be 0.47, considering the differences in the medical environment, including the inclusion criteria. With this assumption, the number of patients required to obtain a power of ≥80% using a 2-sample t-test at a 2-sided significance level of 0.05 was calculated to be 74 patients for each group. Assuming that the dropout rate during the treatment period will be approximately 5%, the enrollment of 80 patients per group (240 patients in total) was estimated as the target sample size.  **MMRM analysis**  The MMRM did not assume a specific covariance structure. If the algorithm did not converge in the above model, Heterogeneous AR(1), Heterogeneous Compound Symmetry, Compound Symmetry, and Variance Component were selected, in this order, as the covariance structure for the MMRM. MMRM was used to compare ISI total score, SF-36 summary score or 8-domain score, HAMD‑17 score, anxiety, Bech-6, Maier-6, and insomnia symptoms.  **Weight of IPW-GEE**  For each visit weight, the inverse of the propensity score, which represents the conditional observation probability of the response up to that visit, was used. The conditional observation probability of the response at each visit was estimated by a logistic regression model with the intervention group, time point, and response at the previous time point as explanatory variables and whether or not a response is observed at each visit as the dependent variable. If there is a missing measurement at a time point that is not consecutive during the study period, the observed response one time point before the missing time point was used as the explanatory variable instead. If there is no missing measurement, the conditional observation probability was set to 1. The conditional observation probability of the response up to the relevant visit was then calculated by the cumulative product of the estimated conditional observation probabilities. |

^†^The following substance-use disorders are specified in DSM-5: alcohol, cannabis, hallucinogens (phencyclidine and other hallucinogens), inhalants, opioids, sedatives, hypnotics, anxiolytics, and stimulants (amphetamines, cocaine, or other stimulants).

^‡^If a participant met all of the following criteria, the participant was suspected of having sleep apnea. The participant was

to be excluded unless the suspicion was ruled out.

- Patients whose score of JESS was ≥11, and it was considered not attributable to major depressive disorder by the investigator or subinvestigator.
- BMI of ≥30.

^§^Refer to Grade 3 described in the “Criteria for Seriousness/Severity Grading of Adverse Drug Reactions” (Notification No. 80 of the Safety Division, Pharmaceutical Affairs Bureau in Japan, dated 29 Jun 1992)

BMI, body mass index; C-SSRS, Columbia-Suicide Severity Rating Scale; DSM-5, Diagnostic and Statistical Manual of Mental Disorders 5th edition; ECG, electrocardiography; FEV1, forced expiratory volume in 1 second; HAMD-17, 17-item Hamilton Depression Rating Scale; ICF, informed consent form; JESS, Japanese version of the Epworth Sleepiness Scale; MDD, major depressive disorder; MGH ATRQ, Massachusetts General Hospital Antidepressant Treatment Response Questionnaire; M.I.N.I., Mini-International Neuropsychiatric Interview; PHQ-9, Patient Health Questionnaire-9; QTc, corrected QT interval; QTcF, corrected QT interval using Fridericia’s formula; SD, standard deviation; TRD, treatment-resistant depression.

**Table S2****.** Discontinuation of study drug and patient discontinuation/withdrawal criteria

| **Discontinuation of study intervention**  In rare instances, it may be necessary for a participant to permanently discontinue (definitive discontinuation) study drug. If study drug is definitively discontinued, the termination visit should be performed as far as possible. |
| --- |
| **Liver chemistry stopping criteria**  The investigator or subinvestigator will consider whether to continue the study due to a liver function test abnormality if a participant meets any of the conditions shown in the figure below or if the investigator or subinvestigator determines that it is in the best interest of the participant even if the abnormality does not meet any of the stopping criteria.  **Figure Liver chemistry stopping criteria and algorithm for enhancement of monitoring**  Continuation of the study  Review whether or not to continue the study  Plus bilirubin ≥2 ⨯ ULN (>35% direct bilirubin) or INR >1.5 (if measured)^†^  ALT ≥3 ⨯ ULN  ALT ≥5 ⨯ ULN  ALT ≥3 ⨯ ULN with symptoms of hepatic disorder or hypersensitivity  ALT ≥3 ⨯ ULN with weekly monitoring allowed for 4 weeks  **No**  **Yes**  **Yes**  **Yes**  **Yes**  **No**  **Yes**  ALT ≥3 ⨯ ULN persisting for 4 weeks or meeting the stopping criteria  **Yes**  **No**  **Yes**   - “Suggested actions and follow-up assessments” should be referred. - Report as an SAE if: ALT ≥3 ⨯ ULN and bilirubin ≥2 ⨯ ULN (>35% direct bilirubin) or ALT ≥3 ⨯ ULN and INR >1.5, if INR measured.   ^†^This INR threshold is not applicable to participants receiving anticoagulants.”  **No**  **No**  **No**   - If the participant is monitored weekly,: “suggested actions and follow-up assessments” should be referred.   ALT, alanine aminotransferase; INR, international normalized ratio; SAE, serious adverse event; ULN, upper limit of normal.   \| **Liver chemistry stopping criteria** \| \| \| \| --- \| --- \| --- \| \| ALT \| ALT ≥5 × ULN \| \| \| Duration of increase in ALT \| ALT ≥3 × ULN persists for ≥4 weeks \| \| \| Bilirubin^†, ‡^ \| ALT ≥3 × ULN and bilirubin ≥2 × ULN (>35% direct bilirubin) \| \| \| INR^‡^ \| ALT ≥3 × ULN and INR >1.5, if INR measured \| \| \| Cannot monitor \| ALT ≥3 × ULN and cannot be monitored weekly for 4 weeks \| \| \| Symptomatic^§^ \| ALT ≥3 × ULN associated with symptoms (new or worsening) believed to be related to hepatic disorder or hypersensitivity \| \| \| **Suggested actions and follow-up assessments** \| \| \| \| **Actions** \| \| **Follow-up assessments** \| \| - **Immediately** discontinue study drug. - Report to the sponsor **within 24 hours**. - Document the results in the Liver Event Form. If the event also meets the criteria for an SAE, report to the sponsor and complete the eCRF^‡^. - Perform liver chemistry event follow-up assessments. - Monitor the participant until abnormal liver chemistries resolve, stabilize, or return to baseline values (see Monitori/ng below). - **Do not restart/rechallenge** the participant with the study drug unless allowed per protocol and sponsor approval **is granted**. - If restart/rechallenge **not allowed per protocol or not granted**, permanently discontinue the study drug, and continue the participant in the study for any protocol-specified follow-up assessments.   **Monitoring:**  **For ALT ≥3 × ULN and bilirubin ≥2 × ULN or ALT ≥3 × ULN and INR >1.5:**   - Repeat liver chemistries (include ALT, AST, ALP, bilirubin, and INR) and perform liver-event follow-up assessments within **24 hours**. - Monitor the participant twice weekly until abnormal liver chemistries resolve, stabilize, or return to baseline values. - Consultation with a hepatologist is recommended.   **For ALT ≥3 × ULN, bilirubin <2 × ULN and INR ≤1.5:**   - Repeat liver chemistries (include ALT, AST, ALP, bilirubin, and INR) and perform liver-event follow-up assessments within **24 to 72 hours**. - Monitor the participant weekly until abnormal liver chemistries resolve, stabilize, or return to baseline values. \| \| - Viral hepatitis serology^¶^. - Obtain INR and recheck with each liver chemistry assessment until the transaminase values show a downward trend. - Obtain blood sample to determine the following^#^: - Serum CPK and LDH - Fractionate bilirubin if total bilirubin ≥2 × ULN - Obtain complete blood count with differential to assess eosinophilia - If the study is discontinued before the treatment period (Day 15), blood will be collected for the measurement of plasma zuranolone concentration, and the measurement will be conducted^#^. - Record any new or worsening symptoms of hepatic disorder or hypersensitivity on the AE eCRF page. - Record use of concomitant medications on the concomitant medications eCRF page, including acetaminophen, herbal medicines, and other over-the-counter medications. - Record alcohol use on the Liver Event Form.   **For ALT ≥3 × ULN and bilirubin ≥2 × ULN, or ALT ≥3 × ULN and INR >1.5:**   - Antinuclear antibody, anti-smooth muscle antibody, Type 1 anti-liver kidney microsomal antibodies, and quantitative total IgG or gamma globulins. - Serum acetaminophen protein^\|\|^ adducts HPLC assay (quantifies potential acetaminophen contribution to hepatic disease in patients with definite or likely acetaminophen use in the preceding week). - Liver imaging (ultrasound, magnetic resonance, or computed tomography) and/or liver biopsy to evaluate liver function; document in the Liver Event Form. \|   ^†^Serum bilirubin fractionation should be performed if testing is available. If serum bilirubin fractionation is not immediately available, discontinue study drug for the participant if ALT ≥3 × ULN and bilirubin ≥2 × ULN. Additionally, if serum bilirubin fractionation testing is unavailable, record presence of detectable urinary bilirubin on dipstick, indicating direct bilirubin elevations and suggesting hepatic disorder.  ^‡^All events of ALT ≥3 × ULN and bilirubin ≥2 × ULN (>35% direct bilirubin) or ALT ≥3 × ULN and INR >1.5, if measured, must be reported as SAEs (excluding studies of hepatic impairment or cirrhosis). INR measurement is not required, and the threshold value stated will not apply to patients receiving anticoagulants.  ^§^New or worsening symptoms believed to be related to hepatic disorder (e.g., fatigue, nausea, vomiting, right upper quadrant pain or tenderness, and jaundice) or believed to be related to hypersensitivity (e.g., fever, rash, and eosinophilia).  ^¶^Includes Hepatitis A IgM antibody, Hepatitis A surface antigen and Hepatitis B Core Antibody, Hepatitis C RNA, Cytomegalovirus IgM antibody, Epstein-Barr viral capsid antigen IgM antibody (or if unavailable, obtain heterophile antibody of monospot testing), and Hepatitis E IgM antibody.  ^#^Record the date and time of blood sample draw and the date and time of the last dose of study drug prior to the blood sample draw in the eCRF. If the date or time of the last dose is unclear, provide the participant’s best approximation. Instructions for sample handling and shipping are separately provided in the operating procedure.  ^\|\|^James LP, Letzig L, Simpson PM, et al. Pharmacokinetics of Acetaminophen-Adduct in Adults with Acetaminophen Overdose and Acute Liver Failure. Drug Metab Dispos 2009; 37:1779-84  AE, adverse event; ALT, alanine aminotransferase; ALP, alkaline phosphatase; AST, aspartate transaminase; CPK, creatinine phosphokinase; eCRF, electronic case report form; HPLC, high-performance liquid chromatography; Ig, immunoglobulin; INR, international normalized ratio; LDH, lactate dehydrogenase; RNA, ribonucleic acid; SAE, severe adverse event; ULN, upper limit of normal.  **QTc stopping criteria**  If a clinically significant QTc finding is identified at visit 1 or subsequent visits, the investigator or subinvestigator determines whether the participant should continue in the study and whether the management of the participant should be changed. Clinically significant findings include, but are not limited to, changes from baseline in the QTcF. The reading of 12-lead ECG at this time must be recorded in the eCRF. All new clinically significant findings should be reported as AEs.  If the mean of three measurements of 12-lead ECG meets any of the following conditions, the investigator or subinvestigator examines whether the participant should continue in the study.   - QTcF >500 msec - QTcF change from baseline >60 msec   For a participant with bundle branch block, the following stopping criteria should be followed. If the participant meets any of the following criteria, the investigator or subinvestigator examines whether the participant should continue in the study.   - For patients with baseline QTc of <450 msec: QTc of >500 msec - For patients with baseline QTc of 450–480 msec: QTc of ≥530 msec   **Discontinuation of study intervention due to pregnancy**  **Male patients with partners who become pregnant**   - The investigator or subinvestigator will attempt to collect pregnancy information on any male participant’s female partner who becomes pregnant while the male participant is in this study. This applies only to male patients who receive the study drug. - If a female partner of a male participant becomes pregnant, the investigator or subinvestigator will, after obtaining the necessary signed informed consent from the pregnant female partner directly, record pregnancy information on the appropriate form and submit it to the sponsor within 24 hours of learning of the partner’s pregnancy. - The female partner will also be followed to determine the outcome of the pregnancy. Follow-up information on the status of the mother and child will be collected by the investigator or subinvestigator and forwarded to the sponsor. Generally, the follow-up assessments should be conducted within 6–8 weeks following the estimated delivery date. Any termination of the pregnancy will be reported regardless of fetal status (presence or absence of anomalies) or indication for surgery/procedure.   **Female patients who become pregnant**   - Any female participant who becomes pregnant while participating in the study will discontinue the study drug or be withdrawn from the study. - The investigator or subinvestigator collected pregnancy information on any female participant who became pregnant while participating in this study. The initial information was recorded on the appropriate form and submitted to the sponsor within 24 hours of learning of a participant’s pregnancy. - The participant was followed up to determine the outcome of the pregnancy. Follow-up information on the status of the mother and child was collected by the investigator or subinvestigator and forwarded to the sponsor. Generally, the follow-up assessments were conducted within 6–8 weeks following the estimated delivery date. Any termination of the pregnancy was reported regardless of fetal status (presence or absence of anomalies) or indication for surgery/procedure. - While pregnancy itself was not considered to be an AE or SAE, any pregnancy complication or elective termination of a pregnancy for medical reasons was reported as an AE or SAE. - A spontaneous abortion (occurring at < 22 weeks gestational age) or stillbirth (occurring at ≥ 22 weeks gestational age) was considered to be an SAE and was reported as such. - Any post-study pregnancy-related SAE considered reasonably related to the study drug by the investigator or subinvestigator was reported to the sponsor using standard procedures. While the investigator or subinvestigator was not obligated to actively seek this information in former study participants, he/she was allowed to learn of an SAE through spontaneous reporting.   **Discontinuation of study intervention for other reasons**  The investigator or subinvestigator should withdraw a participant from the study drug for any of the following reasons:   - A serious or intolerable AE occurs and the investigator or subinvestigator considers that the participant should be withdrawn - The participant requested withdrawal - The participant is found to be ineligible for the study during the screening period - The participant is found to be ineligible for the study after the initiation of study drug - The participant is lost to follow-up - The participant died - The investigator or subinvestigator considers that the target disease (MDD) requires treatment other than that specified in the study protocol - The investigator or subinvestigator determines that the participant should be withdrawn from the study for other reasons   **Participant discontinuation/withdrawal from the study**   - A participant may withdraw from the study at any time at his/her own request or may be withdrawn at any time at the discretion of the investigator or subinvestigator for safety, behavioral, compliance, or administrative reasons. - If the investigator or subinvestigator considers that treatment to ameliorate the target disease (e.g., initiation of treatment with antidepressants, anxiolytics, hypnotics, or other drugs, or addition of ongoing concomitant therapy) is necessary for a participant, the investigator or subinvestigator should determine withdrawal of the participant from the study and discontinue study drug before the start of the treatment. - If the participant withdraws consent, the sponsor may retain and continue to use any data collected before such a withdrawal of consent. - If a participant withdraws from the study, he/she may request destruction of any samples taken and not tested, and the investigator or subinvestigator must document this in the site study records. - At the time of withdrawal from the study, the participant should visit the study site within three days for termination visit, as far as possible. All patients withdrawn from the study due to AEs should be followed until resolution of AEs, until the investigator or subinvestigator determines that the symptoms have stabilized, until the participant can no longer be contacted, or until the investigator or subinvestigator determines that follow-up is unnecessary. The date of completion or discontinuation and the reason for discontinuation should be recorded in the eCRF.   **Lost to follow-up**  A participant will be considered lost to follow-up if he or she fails to return for scheduled visits and is unable to be contacted by the investigator, subinvestigator, or study coordinator.  The following actions must be taken if a participant fails to return to the clinic for a required study visit:   - The investigator, subinvestigator, or study coordinator must attempt to contact the participant as soon as possible, reschedule the missed visit, counsel the participant on the importance of maintaining the assigned visit schedule, and ascertain whether or not the participant wishes to and/or should continue in the study. - Before a participant is deemed lost to follow-up, the personnel designated by the investigator or subinvestigator must make every effort to regain contact with the participant by phone, etc., and the contact attempts should be documented in the participant’s medical record, etc. - Should the participant continue to be unreachable, he/she will be considered to have withdrawn from the study.   **Prior and concomitant therapy**  **Documentation of prior therapy/concomitant therapy**  The investigator or subinvestigator had to record any medication or vaccine (including over-the-counter or prescription medicines, vitamins, and/or herbal supplements) that the participant received during the study period along with the following information on the eCRF.  For medications prior to baseline, all medications used to treat the target disease that were taken after the onset of the current episode had to be reviewed.   - Reason for use - Dates of administration including start and end dates - Dosage information including dose, route of administration, and frequency   **Restrictions of prior therapy/concomitant therapy**   - The use of the following medications was prohibited from 28 days before baseline to completion of study drug (Day 14): - Strong inhibitors of CYP3A - Strong inducers of CYP3A - The use of the following medications was prohibited from 14 days before baseline to   completion or discontinuation of the study:   - Antidepressants - Anxiolytics - Hypnotics (excluding non-GABA hypnotics) - Antipsychotics - Antiparkinsonian drugs - Antiepileptic drugs   - Mood stabilizers (lithium carbonate, carbamazepine, sodium valproate, lamotrigine, etc.) - Other GABA_A_ receptor modulators or drugs that affect the amount of GABA receptor or GABA - Drugs, Chinese herbal medicines, and supplements indicated for depression, depressive state (including similar expressions such as depressed mood), and mental instability - Therapies using devices such as electroconvulsive therapy or transcranial magnetic therapy - Opioid preparations - The following medications were allowed to be used on an as-needed basis up to twice a week from informed consent to completion or discontinuation of the study. The use on the day before each visit was prohibited. - Non-GABA hypnotics (ramelteon, suvorexant, lemborexant, etc.) - Drugs and Chinese herbal medicines used for the treatment of insomnia that do not correspond to the above prohibited concomitant medications (antihistamines, etc.) - Addition of new therapies for the treatment of MDD was prohibited from informed consent to completion or discontinuation of the study. Therapy that had been ongoing for at least 14 days prior to baseline was allowed to be continued but had to remain consistent throughout the study. |
| AE, adverse event; CYP, cytochrome P450; ECG, electrocardiography; eCRF, electronic case report form; GABA, γ-aminobutyric acid; MDD, major depressive disorder; QTc, corrected QT interval; QTcF, corrected QT interval using Fridericia’s formula. |

**Table S3.** Analysis of CGI-S by timepoint (FAS)

| Time point (planned day) | Treatment group | n^†^ | “Normal, not at all ill” or “borderline mentally ill” n^‡^ (%) | Vs placebo | | |
| --- | --- | --- | --- | --- | --- | --- |
|  |  |  |  | Adjusted odds ratio [95% CI] | | *P* |
| 3 | Placebo | 81 | 0 | |  |  |
|  | Zuranolone 20 mg | 84 | 0 | | --- | --- |
|  | Zuranolone 30 mg | 82 | 0 | | --- | --- |
| 8 | Placebo | 82 | 1 (1.2) | |  |  |
|  | Zuranolone 20 mg | 84 | 2 (2.4) | | 2.00 [0.18, 22.58] | 0.5734 |
|  | Zuranolone 30 mg | 81 | 2 (2.5) | | 2.07 [0.18, 23.32] | 0.5568 |
| 15 | Placebo | 82 | 2 (2.4) | |  |  |
|  | Zuranolone 20 mg | 81 | 6 (7.4) | | 3.21 [0.63, 16.35] | 0.1604 |
|  | Zuranolone 30 mg | 80 | 6 (7.5) | | 3.28 [0.64, 16.77] | 0.1530 |
| 22 | Placebo | 79 | 8 (10.1) | |  |  |
|  | Zuranolone 20 mg | 81 | 4 (4.9) | | 0.46 [0.13, 1.61] | 0.2264 |
|  | Zuranolone 30 mg | 77 | 10 (13.0) | | 1.33 [0.50, 3.55] | 0.5705 |
| 29 | Placebo | 79 | 12 (15.2) | |  |  |
|  | Zuranolone 20 mg | 75 | 10 (13.3) | | 0.87 [0.35, 2.16] | 0.7640 |
|  | Zuranolone 30 mg | 76 | 11 (14.5) | | 0.96 [0.39, 2.33] | 0.9256 |
| 36 | Placebo | 78 | 13 (16.7) | |  |  |
|  | Zuranolone 20 mg | 75 | 14 (18.7) | | 1.15 [0.50, 2.65] | 0.7389 |
|  | Zuranolone 30 mg | 74 | 12 (16.2) | | 0.95 [0.40, 2.25] | 0.9081 |
| 43 | Placebo | 74 | 12 (16.2) | |  |  |
|  | Zuranolone 20 mg | 75 | 15 (20.0) | | 1.33 [0.57, 3.09] | 0.5038 |
|  | Zuranolone 30 mg | 71 | 15 (21.1) | | 1.39 [0.60, 3.23] | 0.4409 |
| 50 | Placebo | 74 | 12 (16.2) | |  |  |
|  | Zuranolone 20 mg | 72 | 12 (16.7) | | 0.95 [0.40, 2.24] | 0.9028 |
|  | Zuranolone 30 mg | 72 | 16 (22.2) | | 1.37 [0.60, 3.11] | 0.4497 |
| 57 | Placebo | 71 | 13 (18.3) | |  |  |
|  | Zuranolone 20 mg | 70 | 12 (17.1) | | 0.92 [0.39, 2.21] | 0.8597 |
|  | Zuranolone 30 mg | 70 | 16 (22.9) | | 1.33 [0.58, 3.02] | 0.4986 |

^†^Number of patients with nonmissing CGI-S score at the visit.

^‡^Number of patients with a “normal, not at all ill” or “borderline mentally ill” of CGI-S.

The denominator of the percentage is the number of patients with nonmissing CGI-S score at the visit.

IPW-GEE analysis. If the model is not converged, the earliest time point will be excluded from the model, and “---” will be shown at the timepoint.

Fixed effect: treatment group, time point, interaction effect (treatment group and time point).

Covariate: baseline value of CGI-S score, sex (male, female).

Working correlation structure: independent.

CGI-S, Clinical Global Impression–Severity of Illness; CI, confidence interval; FAS, full analysis set; IPW-GEE, inverse probability-weighted generalized estimating equation.

**Table S4.** Changes from baseline in ISI total score by timepoint (FAS)

| Time point  (Planned day) | Treatment group | Observed value | | Change from baseline LS mean (SE) | Vs Placebo | |
| --- | --- | --- | --- | --- | --- | --- |
|  |  | Mean (SD) | |  | Difference of LS mean [95% CI] | *P* |
| 1 | Placebo | 82 | 18.28 (4.65) | --- |  |  |
|  | Zuranolone 20 mg | 85 | 17.69 (5.04) | --- | --- | --- |
|  | Zuranolone 30 mg | 82 | 17.06 (5.04) | --- | --- | --- |
| 3 | Placebo | 81 | 17.95 (4.81) | −0.24 (0.38) |  |  |
|  | Zuranolone 20 mg | 84 | 16.39 (5.46) | −1.28 (0.37) | −1.04 [−2.08, 0.01] | 0.0513 |
|  | Zuranolone 30 mg | 81 | 15.48 (5.65) | −1.64 (0.38) | −1.40 [−2.46, −0.34] | 0.0095 |
| 8 | Placebo | 82 | 16.55 (5.92) | −1.59 (0.51) |  |  |
|  | Zuranolone 20 mg | 84 | 14.58 (5.77) | −3.11 (0.51) | −1.52 [−2.94, −0.10] | 0.0357 |
|  | Zuranolone 30 mg | 81 | 13.95 (6.08) | −3.15 (0.52) | −1.56 [−2.99, −0.12] | 0.0339 |
| 15 | Placebo | 82 | 15.49 (6.34) | −2.65 (0.60) |  |  |
|  | Zuranolone 20 mg | 80 | 12.60 (6.13) | −4.65 (0.60) | −2.00 [−3.67, −0.34] | 0.0187 |
|  | Zuranolone 30 mg | 80 | 12.45 (6.48) | −4.64 (0.61) | −1.99 [−3.67, −0.31] | 0.0204 |
| 22 | Placebo | 79 | 14.57 (6.81) | −3.46 (0.59) |  |  |
|  | Zuranolone 20 mg | 81 | 13.17 (6.22) | −4.29 (0.58) | −0.84 [−2.46, 0.79] | 0.3119 |
|  | Zuranolone 30 mg | 77 | 13.21 (6.40) | −4.02 (0.59) | −0.57 [−2.21, 1.08] | 0.4986 |
| 29 | Placebo | 79 | 14.71 (7.11) | −3.32 (0.62) |  |  |
|  | Zuranolone 20 mg | 75 | 13.31 (5.93) | −3.96 (0.62) | −0.64 [−2.37, 1.09] | 0.4672 |
|  | Zuranolone 30 mg | 77 | 13.19 (6.66) | −4.06 (0.63) | −0.74 [−2.48, 1.00] | 0.4008 |
| 36 | Placebo | 78 | 14.37 (7.10) | −3.62 (0.63) |  |  |
|  | Zuranolone 20 mg | 74 | 12.84 (6.18) | −4.62 (0.63) | −1.00 [−2.75, 0.74] | 0.2586 |
|  | Zuranolone 30 mg | 74 | 12.23 (6.03) | −4.78 (0.63) | −1.17 [−2.92, 0.59] | 0.1912 |
| 43 | Placebo | 74 | 13.43 (7.05) | −4.13 (0.64) |  |  |
|  | Zuranolone 20 mg | 75 | 12.81 (5.98) | −4.57 (0.64) | −0.44 [−2.22, 1.35] | 0.6322 |
|  | Zuranolone 30 mg | 71 | 12.59 (6.83) | −4.66 (0.65) | −0.53 [−2.33, 1.28] | 0.5642 |
| 50 | Placebo | 74 | 13.77 (6.89) | −4.03 (0.66) |  |  |
|  | Zuranolone 20 mg | 72 | 12.28 (6.21) | −4.77 (0.66) | −0.73 [−2.58, 1.11] | 0.4339 |
|  | Zuranolone 30 mg | 72 | 12.04 (6.88) | −5.18 (0.67) | −1.14 [−3.00, 0.71] | 0.2263 |
| 57 | Placebo | 72 | 13.76 (7.14) | −3.68 (0.65) |  |  |
|  | Zuranolone 20 mg | 70 | 12.26 (6.37) | −4.86 (0.65) | −1.18 [−2.98, 0.63] | 0.2002 |
|  | Zuranolone 30 mg | 70 | 12.49 (6.79) | −4.86 (0.66) | −1.17 [−2.99, 0.65] | 0.2051 |

Statistical methods: mixed-effects model for repeated measures analysis

Fixed effect: treatment group, time point, interaction effect (treatment group and time point).

Covariate: baseline value of ISI total scores, sex (male, female).

Covariance structure: unstructured.

CI, confidence interval; FAS, full analysis set; ISI, Insomnia Severity Index; LS, least square; SD, standard deviation; SE, standard error.

**Table S5.** Incidence of treatment-related AEs during the study period (safety population)

| System organ class  - Preferred term | Placebo N=82  n (%) | Zuranolone 20 mg  N=85 n (%) | Zuranolone 30 mg  N=82 n (%) |
| --- | --- | --- | --- |
| Patients with any treatment-related AEs | 17 (20.7) | 27 (31.8) | 27 (32.9) |
| Psychiatric disorders | 1 (1.2) | 2 (2.4) | 1 (1.2) |
| - Abnormal dreams | 0 | 1 (1.2) | 0 |
| - Insomnia | 0 | 1 (1.2) | 0 |
| - Frustration tolerance decreased | 0 | 0 | 1 (1.2) |
| - Nightmare | 1 (1.2) | 0 | 0 |
| Nervous system disorders | 9 (11.0) | 19 (22.4) | 23 (28.0) |
| - Somnolence | 3 (3.7) | 9 (10.6) | 17 (20.7) |
| - Dizziness | 3 (3.7) | 8 (9.4) | 8 (9.8) |
| - Headache | 3 (3.7) | 3 (3.5) | 1 (1.2) |
| - Dizziness postural | 0 | 1 (1.2) | 0 |
| - Sedation | 0 | 1 (1.2) | 0 |
| - Tremor | 0 | 0 | 1 (1.2) |
| Ear and labyrinth disorders | 1 (1.2) | 0 | 0 |
| - Vertigo | 1 (1.2) | 0 | 0 |
| Cardiac disorders | 0 | 1 (1.2) | 0 |
| - Palpitations | 0 | 1 (1.2) | 0 |
| Gastrointestinal disorders | 6 (7.3) | 6 (7.1) | 3 (3.7) |
| - Constipation | 1 (1.2) | 2 (2.4) | 2 (2.4) |
| - Nausea | 1 (1.2) | 2 (2.4) | 1 (1.2) |
| - Diarrhoea | 0 | 1 (1.2) | 1 (1.2) |
| - Dry mouth | 1 (1.2) | 1 (1.2) | 0 |
| - Abdominal discomfort | 1 (1.2) | 0 | 0 |
| - Abdominal pain upper | 1 (1.2) | 0 | 0 |
| - Stomatitis | 1 (1.2) | 0 | 0 |
| Skin and subcutaneous tissue disorders | 0 | 1 (1.2) | 2 (2.4) |
| - Eczema | 0 | 0 | 1 (1.2) |
| - Night sweats | 0 | 1 (1.2) | 0 |
| - Urticaria | 0 | 0 | 1 (1.2) |
| Reproductive system and breast disorders | 0 | 0 | 1 (1.2) |
| - Menstruation irregular | 0 | 0 | 1 (1.2) |
| General disorders and administration site conditions | 3 (3.7) | 1 (1.2) | 7 (8.5) |
| - Feeling hot | 0 | 0 | 2 (2.4) |
| - Malaise | 0 | 0 | 2 (2.4) |
| - Thirst | 0 | 0 | 2 (2.4) |
| - Asthenia | 0 | 0 | 1 (1.2) |
| - Feeling abnormal | 2 (2.4) | 1 (1.2) | 0 |
| - Gait disturbance | 0 | 0 | 1 (1.2) |
| - Oedema | 1 (1.2) | 0 | 0 |
| Investigations | 2 (2.4) | 3 (3.5) | 2 (2.4) |
| - Blood creatine phosphokinase increased | 0 | 1 (1.2) | 0 |
| - Electrocardiogram QT prolonged | 0 | 0 | 1 (1.2) |
| - Forced expiratory volume decreased | 0 | 1 (1.2) | 0 |
| - Urine leukocyte esterase positive | 0 | 1 (1.2) | 0 |
| - Blood alkaline phosphatase increased | 0 | 0 | 1 (1.2) |
| - Liver function test abnormal | 1 (1.2) | 0 | 0 |
| - Platelet count decreased | 1 (1.2) | 0 | 0 |

AEs were coded using MedDRA Version 23.0 update.

“During the study” denotes the time window from the start of the treatment period to the end of the follow-up period B.

AE, adverse event; MedDRA, Medical Dictionary for Regulatory Activities.

**Table S6.** Incidence of treatment-related AEs of special interest during the study period (safety population)

| System organ class  - Preferred term | Placebo  N=82 n (%) | Zuranolone 20 mg  N=85 n (%) | Zuranolone 30 mg  N=82 n (%) |
| --- | --- | --- | --- |
| Patients with any treatment-related AEs of | 6 (7.3) | 16 (18.8) | 22 (26.8) |
| special interest |  |  |  |
| Sedation | 0 | 1 (1.2) | 0 |
| Somnolence | 3 (3.7) | 9 (10.6) | 17 (20.7) |
| Dizziness | 3 (3.7) | 8 (9.4) | 8 (9.8) |
| Respiratory failure (SMQ) | 0 | 0 | 0 |
| Drug abuse and dependence (SMQ) | 0 | 0 | 0 |

AEs were coded using MedDRA Version 23.0 update.

“During the study” denotes the time window from the start of the treatment period to the end of the follow-up period B.

AE, adverse event; MedDRA, Medical Dictionary for Regulatory Activities; SMQ, Standardised MedDRA Query.
